# The Effect of the Accelerometer Operating Range on Biomechanical Parameters: Stride Length, Velocity, and Peak Tibial Acceleration during Running

**DOI:** 10.3390/s18010130

**Published:** 2018-01-05

**Authors:** Christian Mitschke, Pierre Kiesewetter, Thomas L. Milani

**Affiliations:** Department of Human Locomotion, Chemnitz University of Technology, 09126 Chemnitz, Germany; pierre.kiesewetter@hsw.tu-chemnitz.de (P.K.); thomas.milani@hsw.tu-chemnitz.de (T.L.M.)

**Keywords:** operating range, accelerometer, stride length, peak tibial acceleration, running velocity, wearable sensors

## Abstract

Previous studies have used accelerometers with various operating ranges (ORs) when measuring biomechanical parameters. However, it is still unclear whether ORs influence the accuracy of running parameters, and whether the different stiffnesses of footwear midsoles influence this accuracy. The purpose of the present study was to systematically investigate the influence of OR on the accuracy of stride length, running velocity, and on peak tibial acceleration. Twenty-one recreational heel strike runners ran on a 15-m indoor track at self-selected running speeds in three footwear conditions (low to high midsole stiffness). Runners were equipped with an inertial measurement unit (IMU) affixed to the heel cup of the right shoe and with a uniaxial accelerometer at the right tibia. Accelerometers (at the tibia and included in the IMU) with a high OR of ±70 g were used as the reference and the data were cut at ±32, ±16, and at ±8 g in post-processing, before calculating parameters. The results show that the OR influenced the outcomes of all investigated parameters, which were not influenced by tested footwear conditions. The lower ORs were associated with an underestimation error for all biomechanical parameters, which increased noticeably with a decreasing OR. It can be concluded that accelerometers with a minimum OR of ±32 g should be used to avoid inaccurate measurements.

## 1. Introduction

With the further advancement of sensor technologies and data analyzing techniques, micro-electro-mechanical sensors (MEMS) have become useful tools for biomechanical research and clinical practice [1]. It has been reported that these wearable motion sensors are an inexpensive alternative to optoelectronic systems and force plates, that they are simple to handle, cost-effective, and are suitable for field measurements [2,3,4,5,6]. When using these sensors, spatio-temporal and kinetic parameters during walking and running can be analyzed in clinical as well as in sportive applications. For kinetic measurements, for example, when investigating the impact loads on lower limbs during running under various conditions (e.g., footwear conditions or the influence of fatigue), the peak tibial acceleration (PTA) was examined by some authors using MEMS [7,8,9,10,11,12,13,14,15,16,17,18,19,20]. Thereby, unidirectional accelerations along the longitudinal axis of the tibia, as well as medio-lateral and anterior-posterior accelerations of the tibia were examined. Furthermore, the determination of stride frequency, of walking or running velocity (runVel), and of stride length (strLen) have also been the focus of research that utilizes MEMS [21,22,23,24,25]. To investigate these biomechanical parameters, individually configured sensors or commercially available inertial measurement units (IMUs: e.g., Shimmer, Achillex, or XSens) were used, which combine accelerometers and gyroscopes. In this context, Provot et al. [26] compared a calibrated industrial accelerometer (considered as the gold standard) to an IMU accelerometer in two tests: (a) on a shaker, and (b) on the distal anteromedial aspect of the subject’s tibia during running at 3.33 m/s. They concluded that IMUs can be used for valid measurements of tibial acceleration during running.

Besides the different sensor types, sensor locations, and the various sampling rates, sensors with considerably different accelerometer operating ranges (ORs) have also been used. When investigating walking or running, some studies used ORs between ±2 and ±70 g, with g being the acceleration of gravity [27,28,29,30,31,32]. However, high accelerations act on the sensor in vertical and anterior–posterior directions during the swing phase and in the first 50 ms after foot strike [11,32]. These accelerations can distinctly exceed the gravitational acceleration of 1 g. For instance, vertical accelerations of 24.62 ± 4.1 g were measured with a heel-mounted IMU accelerometer during heel strike, when running at 3.5 ± 0.1 m/s in a neutral running shoe (PUMA FAAS 500) [32]. If the accelerations exceeded the accelerometer OR, a lower accuracy and an underestimated variability of biomechanical parameters derived from accelerometer signals could result. In this context, Ziebart et al. [33] investigated the influence of accelerometer OR and of a sampling rate on peak acceleration during seven jumping tasks. They used a tri-axial accelerometer (Model 7267A, Endevco Corporation, San Juan Capistrano, CA, USA) with a high OR of ±260 g as the reference and compared the peak accelerations with two commercially available tri-axial accelerometers with an OR of ±6 g (device1: ActiGraph GT3X+, ActiGraph LLC, Pensacola, FL, USA and device2: X6-2mini, Gulf Coast Data Concepts, Waveland, MS, USA). They found that the peak impact acceleration was underestimated by up to 35%. Furthermore, the underestimation error was greater for tasks with a greater impact acceleration.

It is currently still unclear whether, and to what extent, high accelerations can influence the accuracy of running parameters when exceeding the accelerometers’ OR. However, this information is necessary to determine whether differences in the investigated biomechanical parameters strLen, runVel, and in the PTA between conditions are caused by measurement errors due to an accelerometer OR that is too low, or by the investigated conditions themselves.

In addition, the sensor signal characteristics of the IMU accelerometer, which is affixed to the heel cup of a running shoe, can be influenced by the midsole stiffness of footwear [5]. The authors found that a decreasing midsole stiffness resulted in an increasing delay in specific signal characteristics, when determining the time of foot touchdown. At this time, it is still unclear whether the accuracies of strLen and runVel are also influenced by altering acceleration signal characteristics due to a change in midsole stiffness.

Since acceleration variables are related to running injuries, information about whether the ORs influences running parameters is highly relevant and could assist coaches, researchers, and clinicians in selecting the most appropriate accelerometer specification for their investigations.

Therefore, the aim of the present study was to investigate the influence of the accelerometer OR on running parameters when reducing the OR stepwise from ±70 g to ±8 g. We hypothesized that the rapid and short spikes at the beginning of ground contact and the high accelerations during the swing phase influenced the accuracy of strLen (H1), runVel (H2), and PTA (H3) significantly, depending on the footwear conditions. The running parameters were determined based on previously published methods, and accelerometers with a high OR of ±70 g were used as the reference.

## 2. Materials and Methods

### 2.1. Participants

Twenty-one recreational male heel strike runners (age: 24.4 ± 4.2 years; height: 178.2 ± 4.0 cm; weight: 74.1 ± 6.5 kg; running experience: 8.9 ± 3.3 years; training hours: 3.5 ± 1.7 h per week), free of injury for the last six months, participated in this study. High force rising rates can be observed after foot touchdown during rearfoot running [34], which results in a higher PTA for rearfoot runners than for forefoot runners [11]. Therefore, to determine the greatest effect of the reduced OR, only heel strike runners were investigated. This study was approved by the university’s Ethics Committee (V-103-17-HS-CM-Bodenkontakt-25082015), and participants gave written informed consent to their participation in the study.

### 2.2. Test Procedure

During the test, participants wore three different commercially available running shoes, which were provided by our laboratory in men’s UK size 8: Adidas AdiStar (ADIDAS); PUMA Speed 600 (PUMA); Asics Gel Nimbus 12 (ASICS). After an individual warm-up and familiarization with the measurement setup, five repeated trials were recorded on a 15-m indoor track in the three footwear conditions. The footwear conditions were investigated in a randomized order. The running speed was individual and self-selected (on average 3.60 ± 0.4 m/s), however it was constant (range: ±0.1 m/s) for each subject, for all of the 15 trials (three footwear conditions, each having five trials). Running speed was monitored using two light barriers placed 4 meters apart.

### 2.3. Experimental Setup

**Figure 1:**
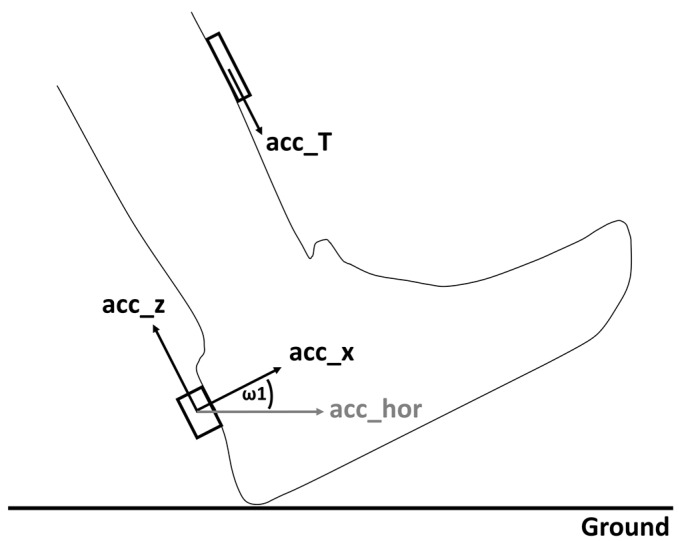
A schematic representation of the sensor setup. Sensitive axes of the accelerometer in the heel cup inertial measurement unit are represented by arrows: horizontal forward direction (acc_x) and vertical acceleration (acc_z). The angular velocity of the shoe was measured in the sagittal (ω1) and frontal plane using a gyroscope. The accelerometer, located at the tibia, measured accelerations along the longitudinal axis of the tibia (acc_T).

An individually configured IMU, combining a biaxial accelerometer (ADXL278, Analog Devices, OR ±70 g) and a biaxial gyroscope (IDG-650, InvenSense, OR ±2000 deg/s), was affixed to the heel cup of the right shoe (Figure 1). Wobble-free sensor fixation was achieved using double-sided adhesive tape and an inelastic strap. The sensitive axes of the IMU accelerometer measured the horizontal—forward direction—(acc_x) and the vertical (acc_z) acceleration of the shoe. The two sensitive axes of the gyroscope measured the angular velocities in the sagittal (ω1), and the frontal planes. Furthermore, to measure accelerations along the longitudinal axis of the tibia (acc_T), double-sided adhesive tape was used to attach a uniaxial lightweight accelerometer (ADXL78, Analog Devices, OR ±70 g) to the shaved skin at the medial aspect mid-distance between the malleolus and the plateau of the right tibia [12,35]. An elastic strap was used to stabilize the accelerometer and to prevent excessive movements due to its own weight. All sensors operated synchronously, and the data were transmitted by cable to a data logger, which recorded these data. The data logger was secured in a waist belt. To avoid any effects from a sampling rate that was too low, the sensor sampling rate was set to 1000 Hz, which exceeds the minimum requirements to measure these parameters accurately [32].

### 2.4. Data Analyses

Data from the sensors were analyzed in post-processing using MATLAB R2016b (MathWorksTM, Natick, MA, USA). Prior to all of the processing steps, the data were filtered using a zero-lag Butterworth low-pass filter (accelerometers: 4th order at 200 Hz; gyroscopes: 4th order at 50 Hz) to remove noise. In the next step, accelerations measured with an OR of ±70 g were cut at ±32, ±16, and at ±8 g when exceeding their respective thresholds. After the data cutting, running parameters were calculated for all four ORs (±70, ±32, ±16, and ±8 g) separately, as described below.

To separate the strides of the right foot in continuous data, the accelerometer signal of the IMU at the heel cup (vertical axis) was 80 Hz zero-lag high pass filtered and the first peak in the filtered signal was defined as the foot touchdown [5,36].

For each stride, the orientation angle of the shoe in the sagittal plane (θ) was calculated using integrated data from ω1. A nulling algorithm was then applied to the orientation angle of the shoe to eliminate errors resulting from integration offset and from drift error [32]. The time of two consecutive flat shoe phases (flat relative to the ground) was detected by finding the lowest absolute angular velocity in ω1 for each stride. The shoe orientation angles at these reference times were used in a linear drift model to eliminate the drift error and the integration offset. Based on Sabatini et al. [21] and Mitschke et al. [32], acc_x, acc_z, and the orientation angle θ were used to calculate the resulting horizontal acceleration (acc_hor) of the right shoe (see Equation (1); Figure 1).

acc_hor = acc_x·cos(θ) − acc_z·sin(θ)
(1)

Subsequently, the horizontal velocity of the shoe (v_x) was calculated using a numerical integration of acc_hor. To eliminate the drift error and the integration offset of the shoe velocity, the velocity of the shoe during the flat shoe phases was assumed to be temporarily equal to zero [37]. Therefore, velocity was reset to zero as the stride start condition and for error back-propagation with a linear drift model [32,38]. The mean running velocity (runVel) was calculated by averaging v_x over time between two consecutive flat shoe phases. Furthermore, the stride length (strLen) between two consecutive flat shoe phases was calculated using integrated data from v_x.

For kinetics, the positive peak tibial acceleration (PTA) was defined as the maximum acceleration value of the accelerometer at the tibia (Figure 1: acc_T) [12,35].

### 2.5. Footwear Conditions

To quantify the shoe midsole stiffness, shoes were tested in a servo-hydraulic testing device (HC10; Zwick GmbH & Co. KG; Ulm, Germany) as described in Schwanitz and Odenwald [39]. Each footwear condition underwent a total of ten load tests of 103 load cycles each. Load cycles were applied to the heel of each running shoe by a spherically shaped stamp (50 mm). The load-time profile was derived from biomechanical measurements of ground reaction force while running at a velocity of 3.5 ± 0.1 m/s [40]. For each footwear condition, midsole deformation at the 101st load cycle was analyzed for each of the ten test sessions. Internal studies have shown that the cycles after the 100th cycle represent reliable results when testing shoes mechanically [41]. The sampling rate was 1000 frames per second, and the data were analyzed using MATLAB R2016b (MathWorksTM, Natick, MA, USA) in post-processing. The midsole stiffness in the rearfoot area between 1000 and 1500 N was calculated for each footwear condition using Equation (2).

(2) $$\mathrm{stiffness}\;=\frac{1500\;N-1000\;N}{{\mathrm{deformation}}_{F=1500\;N}-{\mathrm{deformation}}_{F=1000\;N}}$$

### 2.6. Statistical Analyses

To compare footwear stiffness, the mean and standard deviations (mean ± SD) were calculated. Given that parameters were normally distributed, a one-way analysis of variance (ANOVA) followed by Bonferroni post hoc tests were used to determine whether differences existed between mechanical footwear characteristics. The level of significance was set to an alpha of 0.05. Furthermore, the means of the five trials for each subject and for each footwear condition were used to calculate the group means and the standard deviations (mean ± SD), and the 95% confidence intervals (95% CI) for strLen, runVel, PTA, and all ORs. Due to normal distribution, paired sample t tests were used to compare the reference (±70 g) with the three lower ORs (±32, ±16, and ±8 g) for strLen and runVel for each of the three footwear conditions. In addition, Wilcoxon tests were used to compare the reference with the lower OR for PTA for each footwear condition. A Bonferroni correction was used to adjust the *p*-values (*p* = 0.05/3/3 = 0.006) in relation to the number of comparisons between the investigated OR (*n* = 3) and the investigated biomechanical parameters (*n* = 3). The effect size (Cohen’s *d*) was calculated to quantify the magnitude of differences between the shoe characteristics and the magnitude of differences between the biomechanical parameters examined with different ORs. The coefficients were interpreted as a trivial effect (*d* < 0.2), a small effect (*d* < 0.5), a medium effect (*d* < 0.8), and a large effect (*d* ≥ 0.8) [42]. Additionally, mean differences (MDs), relative mean differences in percent (MD_rel), and root mean square errors (RMSEs) were calculated between the reference OR and each of the lower ORs.

## 3. Results

### 3.1. Footwear Characteristics

All pair-wise comparisons reached statistical significance (*p* < 0.001). Cohen’s *d* showed large effects (*d* ≥ 0.8) in all footwear conditions. The lowest rearfoot stiffness was found for ASICS (156.9 ± 0.1 N/mm) and the highest stiffness was found for ADIDAS (210.3 ± 0.4 N/mm) (Table 1).

### 3.2. Biomechanical Parameters: Stride Length, Running Velocity, and Peak Tibial Acceleration

**Figure 2:**
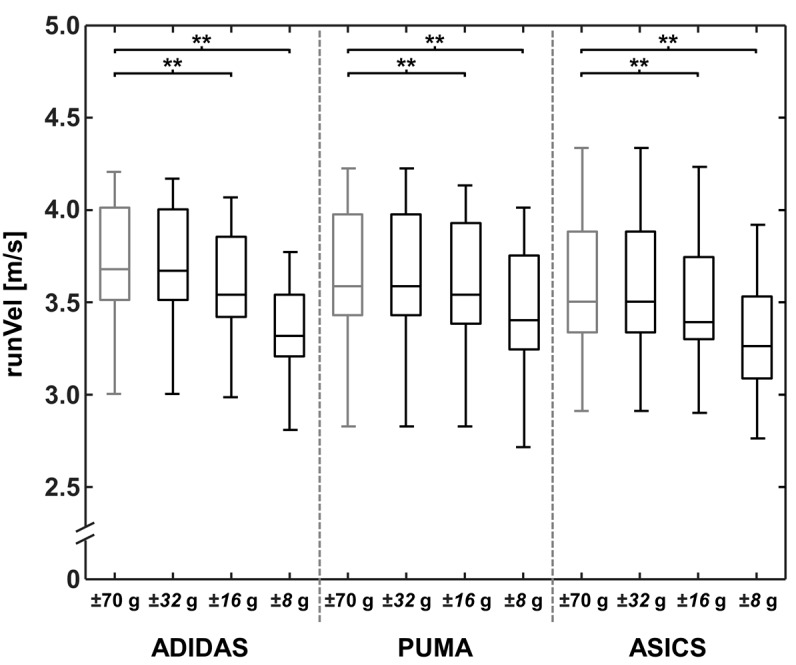
A boxplot of the three footwear conditions for running velocity (runVel), calculated with different accelerometer operating ranges of ±70, ±32, ±16, and ±8 g. Significant differences between stride lengths are marked with ** (*p* < 0.001).

**Figure 3:**
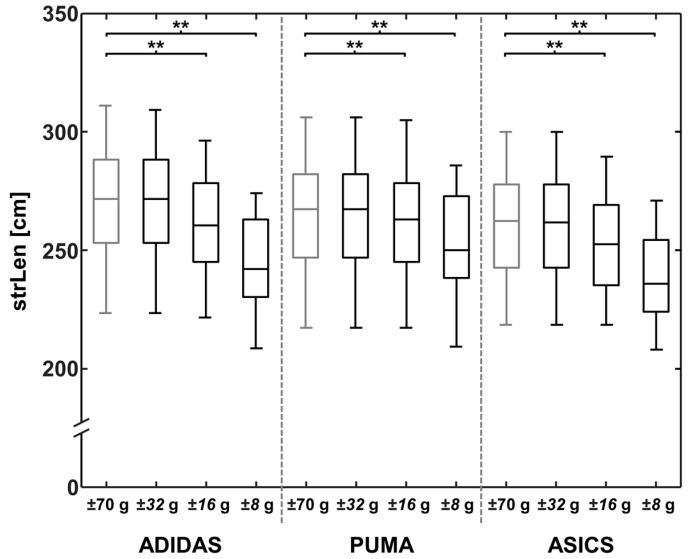
A boxplot of the three footwear conditions for stride length (strLen), calculated with different accelerometer operating ranges of ±70, ±32, ±16, and ±8 g. Significant differences between running velocities are marked with ** (*p* < 0.001).

**Figure 4:**
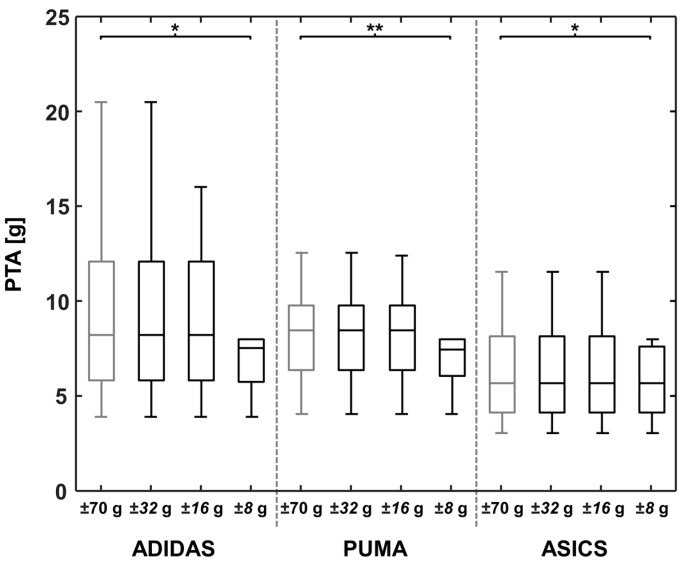
A boxplot of the three footwear conditions for the positive peak tibial acceleration (PTA), determined using different accelerometer operating ranges of ±70, ±32, ±16, and ±8 g. Significant differences between peak tibial accelerations are marked with * (*p* < 0.006) and ** (*p* < 0.001).

For each footwear condition, results of the strLen and the runVel calculations, as well as the PTA values are represented in Figure 2, Figure 3 and Figure 4 for the reference OR and for the lower ORs.

When comparing the OR of ±70 g and ±32 g, no significant differences were found for runVel for all three footwear conditions (Table 2). Distinctly greater differences to the reference were found using the ORs of ±16 g and ±8 g. Setting the OR to ±16 g resulted in significant differences, up to 3.48% (ADIDAS: MD: 0.13 m/s; *p* < 0.001), and large effects (*d* ≥ 0.86) for all three footwear conditions. When using an OR of ±8 g, a significantly lower runVel of up to 9.68% (ADIDAS: MD: 0.36 m/s; *p* < 0.001) was found.

When comparing strLen, calculated using ORs of ±70 g and ±32 g, no significant effects were found for ADIDAS, PUMA, or ASICS (Table 2). When using sensors with a lower OR, strLen calculations resulted in significant differences for all footwear conditions (MD > 2.62 cm; MD_rel > 0.99%; *p* < 0.001), showing large effects for all comparisons (*d* > 0.84). The highest strLen difference between the reduced OR and the reference was found for Adidas. An OR of ±8 g resulted in a 9.68% lower stride length in comparison to the reference OR (MD: 26.15 cm; *p* < 0.001).

No significant differences were found for PTA when comparing an OR of ±70 g and ±32 g, and of ±70 g and ±16 g for all footwear conditions (Table 2). The comparison of the OR ±70 g and ±8 g revealed significant differences for all footwear conditions of at least 9.76% on average (ASICS: MD: 0.61 g), which resulted in a medium effect (*d* = 0.60). The greatest difference between the OR of ±70 g and ±8 g was found for ADIDAS (MD: 2.65 g; MD_rel: 28.17%; *p* = 0.002; *d* = 0.65). In general, we found that with higher midsole stiffnesses, the differences increased between the reference OR and ±16 g, as well as between the reference OR and ±8 g (Figure 4 and Table 2).

## 4. Discussion

The aim of the present study was to investigate the influence of the accelerometer OR on stride length, running velocity, and on peak tibial acceleration when reducing the OR stepwise from ±70 g to ±32, ±16, and ±8 g. Biomechanical parameters were determined based on previously published methods, and accelerometers (attached at the heel cup and at the tibia) with a high OR of ±70 g were used as the reference.

The results of this study revealed that OR influences the outcomes of stride length, running velocity, and peak tibial acceleration, which were not dependent on tested footwear conditions. Lower ORs were associated with an underestimation error for all biomechanical parameters, which increased with decreasing OR. These results confirm hypotheses H1 (strLen), H2 (runVel), and H3 (PTA). Our results show that a sensor OR needs to be carefully considered when interpreting biomechanical parameters of existing investigations and when planning future studies.

**Figure 5:**
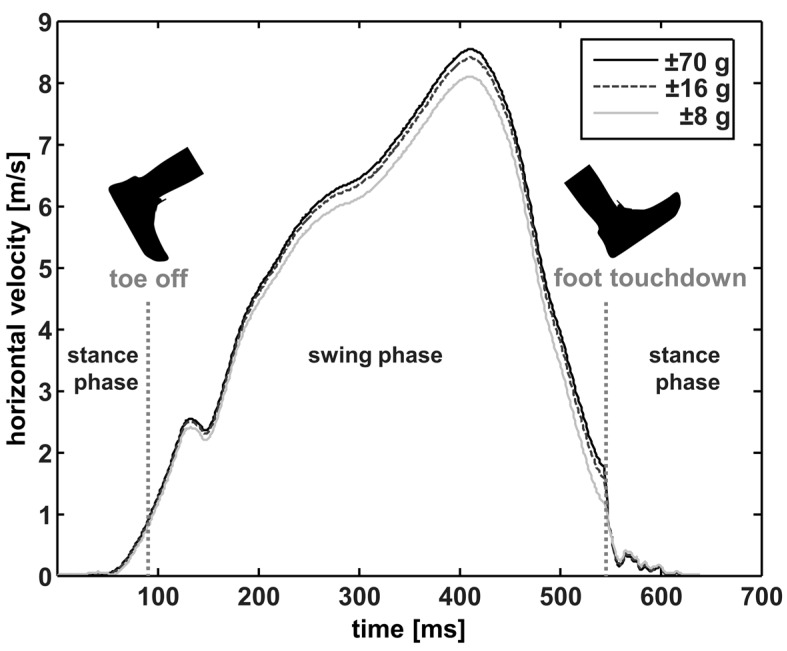
The horizontal velocity (forward direction) of the shoe for one representative stride with an accelerometer operating range of ±70 g (black line), ±16 g (grey dotted line), and of ±8 g (grey line), subdivided in swing phase and foot contact (until toe off, exceeding 2 g in the horizontal acceleration signal [43] and after the foot touchdown [5,36]).

Using accelerometers with an OR of ±32 g resulted in small errors, on average of up to 0.14% for strLen and 0.14% for runVel (ADIDAS). However, insignificant differences and low RMSE values were found for all footwear conditions, and therefore, the observed differences can be considered irrelevant. The accelerations during running that were measured in our study did not critically exceed the ±32 g threshold. However, our results show that when the sensor OR was limited to ±16 g or ±8 g, distinctly higher differences, when compared to the reference, were found for runVel and strLen. Due to the lower vertical and horizontal forward accelerations (set to ±16 g and ±8 g when exceeding the respective thresholds), the resulting horizontal forward velocity of the shoe (v_x) was calculated inaccurately when using the numerical integration of acc_hor. Figure 5 shows v_x for one representative stride. When observing the velocity curve progression, it seems there was a drift error after the numerical integration (frame 120 to 520), which would explain the significantly lower runVel for the OR of ±16 g and ±8 g when compared to the reference. However, to eliminate any drift error and integration offset, the shoe velocity during the flat shoe phases was assumed to be temporarily equal to zero [37]. Therefore, the velocity was reset to zero as the stride start condition and for error back-propagation with a linear drift model [32,38]. As shown in Figure 5, the horizontal forward velocity of the foot was reset to zero in both of the flat shoe phases and no additional offset was observed for these phases. We presume, therefore, that the lower velocities were the result of ORs which were too low and were not due to a drift error.

Additionally, the results show that the stiffnesses of the tested footwear conditions do not appear to noticeably influence the accuracy of runVel. Due to the high impact loads during rearfoot running, we expected that the footwear with the highest midsole stiffness in the rearfoot area (ADIDAS) would show distinctly greater differences between ±70 g and the lower ORs, than the footwear with the lowest stiffness (ASICS). However, whereas ADIDAS revealed the highest differences between the reference OR and the reduced ORs, the ASICS shoe showed higher differences between the reference OR and the reduced ORs than PUMA (medium stiffness) did. Furthermore, similar significant differences (*p* < 0.001) and effect sizes between the reference OR and the OR of ±16 g and ±8 g were found for all of the footwear conditions. Therefore, due to the high accelerations which occurred during the heel strike while running in footwear with differing midsole stiffnesses, sensors with an OR greater than ±32 g should be used to measure these accelerations accurately and to calculate runVel with high accuracy (errors lower than 0.14%).

Despite the different acceleration magnitudes which occurred during heel strike for the three footwear conditions, the calculated velocities (OR: ±70 g) were comparable using the measurements taken by the light barriers (3.60 ± 0.4 m/s): ADIDAS: 3.70 ± 0.3 m/s; PUMA: 3.66 ± 0.4 m/s; ASICS: 3.60 ± 0.3 m/s. Therefore, the method for calculating runVel can be used when the OR is >±32 g.

For strLen, effects due to the lower OR were also observed. StrLen was calculated between two consecutive flat shoe phases using the integrated data from v_x. As expected, lower velocities for the reduced ORs also resulted in shorter strLen. The values of MD_rel and the significant differences between the reference and the reduced ORs, including effect sizes, were similar for strLen and runVel. This is the result of the numerical integration of v_x between two consecutive flat shoe phases when calculating strLen. Additionally, tested footwear conditions did not influence strLen calculations when using an OR of ±32 g. It seems that the critical accelerations in anterior–posterior and in vertical directions do not critically exceed the OR of ±32 g during heel strike, regardless of the midsole stiffness. However, using an OR of ±16 g can lead to strLen differences of 3.49% (MD: 9.43 cm) for each stride when running in the ADIDAS shoe. In contrast, when running in the PUMA shoe, differences of only 0.99% (MD: 2.62 cm) were found. With a further decrease in the OR, the differences between the calculated strLen of PUMA and of ADIDAS increased. When investigating mechanical midsole characteristics of footwear in biomechanical tests (e.g., benchmark tests) using an OR that is too low (<±32 g), errors in the strLen calculation can lead to incorrect conclusions. Therefore, to avoid effects of an OR that is too low, an OR of at least ±32 g should be used to measure strLen as accurately as possible and independently from the midsole stiffness of footwear.

For PTA, none of the subjects in this study generated higher peak tibial accelerations than ±32 g when running in the three provided footwear conditions (MD_rel: 0.0%; RMSE: 0.0 g). However, faster running velocities (e.g., sprint runs), running in shoes with a higher midsole stiffness (e.g., barefoot or minimal shoes), or running barefoot may increase peak tibial acceleration [10,15,44]. In some cases, accelerations could then exceed 32 g and may lead to inaccurate results when measuring with an OR that is too low. Our results for PTA show that accuracy decreased up to 28.17% (ADIDAS: ±8 g) with lower OR. Thereby, we found that a higher midsole stiffness generally decreased the accuracy of PTA. The footwear condition with the stiffest midsole (ADIDAS) showed the greatest PTA for the reference OR (mean ± SD: 9.41 ± 5.3 g), whereas the footwear with the softest midsole configuration (ASICS) showed the lowest PTA (mean ± SD: 6.28 ± 3.0 g). When observing the mean values of PTA for each footwear condition, the minimum OR can be derived from these results.

To accurately determine PTA, a distinctly lower OR (≥±16 g) can be used in contrast to the strLen and runVel (≥±32 g). Due to various influencing factors (e.g., heel fat pad cushioning, foot pronation, shoe midsole deformation, skin displacement, and sensor wobbling), measured accelerations at the tibia were damped and were attenuated in contrast to the wobble-free sensor at the heel cup. Therefore, a distinctly lower OR is necessary for the accelerometer placed at the tibia to determine PTA without a significant loss of information. However, different locations of the accelerometers on the tibia can also influence the temporal and spectral parameters of PTA. Lucas-Cuevas et al. [45] found that a distally placed accelerometer measured a greater PTA and shock attenuation compared to a proximal accelerometer. In our study, the accelerometer was placed at the medial aspect mid-distance between the malleolus and plateau of the right tibia according to Henning et al. [35] and Milani et al. [12]. Therefore, a more distal sensor attachment may require a greater OR than was recommended in our study.

## 5. Conclusions

In literature, various accelerometers with ORs between ±2 g and ±70 g have been used to determine biomechanical parameters, although some studies did not state the OR used. In the present study, the influence of OR on biomechanical parameters was investigated. Our results are important for coaches, researchers, and clinicians in selecting the best accelerometer specification for their future investigations. Our study shows that an accelerometer OR that is set too low results in an inaccurate determination of strLen, runVel, and PTA. Especially in prolonged runs, when investigating a large number of continuous strides (e.g., a marathon [24]), an OR that is too low leads to an underestimation of strLen for each stride, which results in great differences in the total distance. Furthermore, when investigating strLen or PTA variability during prolonged runs in the context of fatigue, a low PTA or strLen variability can be misinterpreted due to an OR that is too low. Additionally, the midsole stiffnesses of the footwear conditions did not influence the accuracy of biomechanical parameters when the OR was ≥±32 g. Furthermore, significant differences and effect sizes between the reference OR and the reduced ORs (±16 g and ±8 g) were similar for all footwear conditions. However, our results show that when investigating mechanical footwear characteristics in biomechanical tests (e.g., benchmark tests), an OR that is too low (for strLen and runVel < ±32 g, for PTA < ±16 g) can lead to an approximation of biomechanical parameters, which leads to incorrect conclusions. Finally, when examining biomechanical parameters during running using accelerometers with an OR lower than ±32 g, these results should be considered carefully.

## Figures and Tables

**Table 1:** Group mean ± standard deviation (SD) for rearfoot stiffness of the three footwear conditions; statistical significance (*p* < 0.05) and large effects (*d* ≥ 0.8) were found in all footwear conditions.

Footwear Condition	Stiffness (N/mm)	
	Mean	SD
ADIDAS	210.3	0.4
PUMA	177.8	0.4
ASICS	156.9	0.1

**Table 2:** Mean differences (MDs), relative mean differences (MD_rel), *p*-values of pair-wise comparisons, effect size (Cohen’s *d*), and root mean square errors (RMSEs) between stride lengths (strLen), running velocities (runVel), and PTA, each determined using different accelerometer operating ranges of ±70, ±32, ±16, and ±8 g.

Footwear Condition	ADIDAS			PUMA			ASICS		
Operating ranges	±70–±32 g	±70–±16 g	±70–±8 g	±70–±32 g	±70–±16 g	±70–±8 g	±70–±32 g	±70–±16 g	±70–±8 g
runVel									
MD (cm)	0.01	0.13	0.36	0.00	0.04	0.18	0.00	0.09	0.29
MD_rel (%)	0.14	3.48	9.68	0.00	1.00	4.88	0.05	2.64	8.18
*p*	0.029	<0.001	<0.001	1	<0.001	<0.001	0.110	<0.001	<0.001
Cohen’s *d*	-	0.86	0.95	-	0.84	0.92	-	0.85	0.94
RMSE (cm)	0.01	0.11	0.27	0.00	0.03	0.14	0.00	0.08	0.22
strLen									
MD (m/s)	0.38	9.43	26.15	0.00	2.62	12.87	0.12	6.92	21.22
MD_rel (%)	0.14	3.49	9.68	0.00	0.99	4.85	0.05	2.67	8.17
*p*	0.024	<0.001	<0.001	1	<0.001	<0.001	0.096	<0.001	<0.001
Cohen’s *d*	-	0.86	0.95	-	0.84	0.92	-	0.85	0.94
RMSE (m/s)	0.57	7.75	19.44	0.00	2.19	9.88	0.23	5.75	15.98
PTA									
MD (g)	0.00	0.59	2.65	0.00	0.31	2.08	0.00	0.00	0.61
MD_rel (%)	0.00	6.25	28.17	0.00	3.48	23.02	0.00	0.04	9.76
*p*	1	0.043	0.002	1	0.109	<0.001	1	0.317	0.005
Cohen’s *d*	-	-	0.65	-	-	0.77	-	-	0.60
RMSE (g)	0.00	1.23	3.39	0.00	0.83	2.72	0.00	0.01	1.13
